# Supplementary material for: HIVprotI: an integrated web based platform for prediction and design of HIV proteins inhibitors
Source: J Cheminform. 2018 Mar 9;10:12. doi: 10.1186/s13321-018-0266-y (PMC5845081; doi:10.1186/s13321-018-0266-y)
Supplement: Supplementary file 2 — Additional file 2. Source code of HIVProtI web server. [file 13321_2018_266_MOESM2_ESM.zip › HIVprotI_Source-code/hivprotI/weka-3-6-6/doc/weka/gui/boundaryvisualizer/BoundaryPanel.html]

BoundaryPanel


---


|  |  |  |
| --- | --- | --- |
| **Overview** \| **Package** \| **Class** \| **Tree** \| **Deprecated** \| **Index** \| **Help** \| **Weka's home** | |  |
| PREV CLASS   **NEXT CLASS** | **FRAMES**    **NO FRAMES**     **All Classes** |  |
| SUMMARY: NESTED \| FIELD \| CONSTR \| METHOD | DETAIL: FIELD \| CONSTR \| METHOD |  |


---


## weka.gui.boundaryvisualizer Class BoundaryPanel

```
java.lang.Object
  java.awt.Component
      java.awt.Container
          javax.swing.JComponent
              javax.swing.JPanel
                  weka.gui.boundaryvisualizer.BoundaryPanel
```

**All Implemented Interfaces:**: java.awt.image.ImageObserver, java.awt.MenuContainer, java.io.Serializable, javax.accessibility.Accessible

**Direct Known Subclasses:**: BoundaryPanelDistributed

---

``` public class BoundaryPanel extends javax.swing.JPanel ```

BoundaryPanel. A class to handle the plotting operations
associated with generating a 2D picture of a classifier's decision
boundaries.

**Since:**
:   1.0

**Version:**
:   $Revision: 7059 $

**Author:**
:   Mark Hall

**See Also:**: `JPanel`, Serialized Form

---

**Nested Class Summary**

**Nested classes/interfaces inherited from class javax.swing.JComponent**

`javax.swing.JComponent.AccessibleJComponent`

**Nested classes/interfaces inherited from class java.awt.Component**

`java.awt.Component.BaselineResizeBehavior`


| **Field Summary** | |
| --- | --- |
| `static java.awt.Color[]` | `DEFAULT_COLORS`             default colours for classes |
| `static double` | `REMOVE_POINT_RADIUS`             The distance we can click away from a point in the GUI and still remove it. |

**Fields inherited from class javax.swing.JComponent**

`TOOL_TIP_TEXT_KEY, UNDEFINED_CONDITION, WHEN_ANCESTOR_OF_FOCUSED_COMPONENT, WHEN_FOCUSED, WHEN_IN_FOCUSED_WINDOW`

**Fields inherited from class java.awt.Component**

`BOTTOM_ALIGNMENT, CENTER_ALIGNMENT, LEFT_ALIGNMENT, RIGHT_ALIGNMENT, TOP_ALIGNMENT`

**Fields inherited from interface java.awt.image.ImageObserver**

`ABORT, ALLBITS, ERROR, FRAMEBITS, HEIGHT, PROPERTIES, SOMEBITS, WIDTH`


**Constructor Summary**

`BoundaryPanel(int panelWidth,
int panelHeight)`
  
          Creates a new `BoundaryPanel` instance.


| **Method Summary** | |
| --- | --- |
| `void` | `addActionListener(java.awt.event.ActionListener newListener)`             Register a listener to be notified when plotting completes |
| `void` | `addMouseListener(java.awt.event.MouseListener l)`             Adds a mouse listener. |
| `void` | `addTrainingInstance(Instance instance)`             Adds a training instance to the visualization dataset. |
| `void` | `addTrainingInstanceFromMouseLocation(int mouseX, int mouseY, int classAttIndex, double classValue)`             Adds a training instance to our dataset, based on the coordinates of the mouse on the panel. |
| `void` | `computeMinMaxAtts()`             Set up the bounds of our graphic based by finding the smallest reasonable area in the instance space to surround our data points. |
| `FastVector` | `getColors()`             Get the current vector of Color objects used for the classes |
| `double` | `getGeneratorSamplesBase()`             Get the base used for computing the number of samples to obtain from each generator |
| `double` | `getMaxXBound()`             Gets the maximum x-coordinate bound, in training-instance units (not mouse coordinates). |
| `double` | `getMaxYBound()`             Gets the maximum x-coordinate bound, in training-instance units (not mouse coordinates). |
| `double` | `getMinXBound()`             Gets the minimum x-coordinate bound, in training-instance units (not mouse coordinates). |
| `double` | `getMinYBound()`             Gets the minimum y-coordinate bound, in training-instance units (not mouse coordinates). |
| `int` | `getNumSamplesPerRegion()`             Get the number of points to sample from a region (fixed dimensions). |
| `boolean` | `getPlotTrainingData()`             Returns true if training data is to be superimposed |
| `static void` | `main(java.lang.String[] args)`             Main method for testing this class |
| `void` | `plotTrainingData()`             Render the training points on-screen. |
| `void` | `removeActionListener(java.awt.event.ActionListener removeListener)`             Remove a listener |
| `void` | `removeAllInstances()`             Deletes all training instances from our dataset. |
| `void` | `removeTrainingInstanceFromMouseLocation(int mouseX, int mouseY)`             Removes a single training instance from our dataset, if there is one that is close enough to the specified mouse location. |
| `void` | `replot()`             Quickly replot the display using cached probability estimates |
| `void` | `setClassifier(Classifier classifier)`             Set the classifier to use. |
| `void` | `setColors(FastVector colors)`             Set a vector of Color objects for the classes |
| `void` | `setDataGenerator(DataGenerator dataGenerator)`             Set the data generator to use for generating new instances |
| `void` | `setGeneratorSamplesBase(double ksb)`             Set the base for computing the number of samples to obtain from each generator. |
| `void` | `setNumSamplesPerRegion(int num)`             Set the number of points to uniformly sample from a region (fixed dimensions). |
| `void` | `setPlotTrainingData(boolean pg)`             Set whether to superimpose the training data plot |
| `void` | `setTrainingData(Instances trainingData)`             Set the training data to use |
| `void` | `setXAttribute(int xatt)`             Set the x attribute index |
| `void` | `setYAttribute(int yatt)`             Set the y attribute index |
| `void` | `start()`             Start the plotting thread |
| `void` | `startPlotThread()`             Starts the plotting thread. |
| `void` | `stopPlotting()`             Stop the plotting thread |

**Methods inherited from class javax.swing.JPanel**

`getAccessibleContext, getUI, getUIClassID, setUI, updateUI`

**Methods inherited from class javax.swing.JComponent**

`addAncestorListener, addNotify, addVetoableChangeListener, computeVisibleRect, contains, createToolTip, disable, enable, firePropertyChange, firePropertyChange, firePropertyChange, getActionForKeyStroke, getActionMap, getAlignmentX, getAlignmentY, getAncestorListeners, getAutoscrolls, getBaseline, getBaselineResizeBehavior, getBorder, getBounds, getClientProperty, getComponentPopupMenu, getConditionForKeyStroke, getDebugGraphicsOptions, getDefaultLocale, getFontMetrics, getGraphics, getHeight, getInheritsPopupMenu, getInputMap, getInputMap, getInputVerifier, getInsets, getInsets, getListeners, getLocation, getMaximumSize, getMinimumSize, getNextFocusableComponent, getPopupLocation, getPreferredSize, getRegisteredKeyStrokes, getRootPane, getSize, getToolTipLocation, getToolTipText, getToolTipText, getTopLevelAncestor, getTransferHandler, getVerifyInputWhenFocusTarget, getVetoableChangeListeners, getVisibleRect, getWidth, getX, getY, grabFocus, isDoubleBuffered, isLightweightComponent, isManagingFocus, isOpaque, isOptimizedDrawingEnabled, isPaintingForPrint, isPaintingTile, isRequestFocusEnabled, isValidateRoot, paint, paintImmediately, paintImmediately, print, printAll, putClientProperty, registerKeyboardAction, registerKeyboardAction, removeAncestorListener, removeNotify, removeVetoableChangeListener, repaint, repaint, requestDefaultFocus, requestFocus, requestFocus, requestFocusInWindow, resetKeyboardActions, reshape, revalidate, scrollRectToVisible, setActionMap, setAlignmentX, setAlignmentY, setAutoscrolls, setBackground, setBorder, setComponentPopupMenu, setDebugGraphicsOptions, setDefaultLocale, setDoubleBuffered, setEnabled, setFocusTraversalKeys, setFont, setForeground, setInheritsPopupMenu, setInputMap, setInputVerifier, setMaximumSize, setMinimumSize, setNextFocusableComponent, setOpaque, setPreferredSize, setRequestFocusEnabled, setToolTipText, setTransferHandler, setVerifyInputWhenFocusTarget, setVisible, unregisterKeyboardAction, update`

**Methods inherited from class java.awt.Container**

`add, add, add, add, add, addContainerListener, addPropertyChangeListener, addPropertyChangeListener, applyComponentOrientation, areFocusTraversalKeysSet, countComponents, deliverEvent, doLayout, findComponentAt, findComponentAt, getComponent, getComponentAt, getComponentAt, getComponentCount, getComponents, getComponentZOrder, getContainerListeners, getFocusTraversalKeys, getFocusTraversalPolicy, getLayout, getMousePosition, insets, invalidate, isAncestorOf, isFocusCycleRoot, isFocusCycleRoot, isFocusTraversalPolicyProvider, isFocusTraversalPolicySet, layout, list, list, locate, minimumSize, paintComponents, preferredSize, printComponents, remove, remove, removeAll, removeContainerListener, setComponentZOrder, setFocusCycleRoot, setFocusTraversalPolicy, setFocusTraversalPolicyProvider, setLayout, transferFocusBackward, transferFocusDownCycle, validate`

**Methods inherited from class java.awt.Component**

`action, add, addComponentListener, addFocusListener, addHierarchyBoundsListener, addHierarchyListener, addInputMethodListener, addKeyListener, addMouseMotionListener, addMouseWheelListener, bounds, checkImage, checkImage, contains, createImage, createImage, createVolatileImage, createVolatileImage, dispatchEvent, enable, enableInputMethods, firePropertyChange, firePropertyChange, firePropertyChange, firePropertyChange, firePropertyChange, getBackground, getBounds, getColorModel, getComponentListeners, getComponentOrientation, getCursor, getDropTarget, getFocusCycleRootAncestor, getFocusListeners, getFocusTraversalKeysEnabled, getFont, getForeground, getGraphicsConfiguration, getHierarchyBoundsListeners, getHierarchyListeners, getIgnoreRepaint, getInputContext, getInputMethodListeners, getInputMethodRequests, getKeyListeners, getLocale, getLocation, getLocationOnScreen, getMouseListeners, getMouseMotionListeners, getMousePosition, getMouseWheelListeners, getName, getParent, getPeer, getPropertyChangeListeners, getPropertyChangeListeners, getSize, getToolkit, getTreeLock, gotFocus, handleEvent, hasFocus, hide, imageUpdate, inside, isBackgroundSet, isCursorSet, isDisplayable, isEnabled, isFocusable, isFocusOwner, isFocusTraversable, isFontSet, isForegroundSet, isLightweight, isMaximumSizeSet, isMinimumSizeSet, isPreferredSizeSet, isShowing, isValid, isVisible, keyDown, keyUp, list, list, list, location, lostFocus, mouseDown, mouseDrag, mouseEnter, mouseExit, mouseMove, mouseUp, move, nextFocus, paintAll, postEvent, prepareImage, prepareImage, remove, removeComponentListener, removeFocusListener, removeHierarchyBoundsListener, removeHierarchyListener, removeInputMethodListener, removeKeyListener, removeMouseListener, removeMouseMotionListener, removeMouseWheelListener, removePropertyChangeListener, removePropertyChangeListener, repaint, repaint, repaint, resize, resize, setBounds, setBounds, setComponentOrientation, setCursor, setDropTarget, setFocusable, setFocusTraversalKeysEnabled, setIgnoreRepaint, setLocale, setLocation, setLocation, setName, setSize, setSize, show, show, size, toString, transferFocus, transferFocusUpCycle`

**Methods inherited from class java.lang.Object**

`equals, getClass, hashCode, notify, notifyAll, wait, wait, wait`

**Field Detail**

### DEFAULT\_COLORS

```
public static final java.awt.Color[] DEFAULT_COLORS
```

:   default colours for classes

---


### REMOVE\_POINT\_RADIUS

```
public static final double REMOVE_POINT_RADIUS
```

:   The distance we can click away from a point in the GUI and still remove it.

    **See Also:**: Constant Field Values


**Constructor Detail**

### BoundaryPanel

```
public BoundaryPanel(int panelWidth,
                     int panelHeight)
```

:   Creates a new `BoundaryPanel` instance.

    **Parameters:**: `panelWidth` - the width in pixels of the panel: `panelHeight` - the height in pixels of the panel


**Method Detail**

### setNumSamplesPerRegion

```
public void setNumSamplesPerRegion(int num)
```

:   Set the number of points to uniformly sample from a region (fixed
    dimensions).

    :   **Parameters:**: `num` - an `int` value

---


### getNumSamplesPerRegion

```
public int getNumSamplesPerRegion()
```

:   Get the number of points to sample from a region (fixed dimensions).

    :   **Returns:**: an `int` value

---


### setGeneratorSamplesBase

```
public void setGeneratorSamplesBase(double ksb)
```

:   Set the base for computing the number of samples to obtain from each
    generator. number of samples = base ^ (# non fixed dimensions)

    :   **Parameters:**: `ksb` - a `double` value

---


### getGeneratorSamplesBase

```
public double getGeneratorSamplesBase()
```

:   Get the base used for computing the number of samples to obtain from
    each generator

    :   **Returns:**: a `double` value

---


### stopPlotting

```
public void stopPlotting()
```

:   Stop the plotting thread

---


### computeMinMaxAtts

```
public void computeMinMaxAtts()
```

:   Set up the bounds of our graphic based by finding the smallest reasonable
    area in the instance space to surround our data points.

---


### start

```
public void start()
           throws java.lang.Exception
```

:   Start the plotting thread

    :   **Throws:**: `java.lang.Exception` - if an error occurs

---


### plotTrainingData

```
public void plotTrainingData()
```

:   Render the training points on-screen.

---


### setTrainingData

```
public void setTrainingData(Instances trainingData)
                     throws java.lang.Exception
```

:   Set the training data to use

    :   **Parameters:**: `trainingData` - the training data **Throws:**: `java.lang.Exception` - if an error occurs

---


### addTrainingInstance

```
public void addTrainingInstance(Instance instance)
```

:   Adds a training instance to the visualization dataset.

---


### addActionListener

```
public void addActionListener(java.awt.event.ActionListener newListener)
```

:   Register a listener to be notified when plotting completes

    :   **Parameters:**: `newListener` - the listener to add

---


### removeActionListener

```
public void removeActionListener(java.awt.event.ActionListener removeListener)
```

:   Remove a listener

    :   **Parameters:**: `removeListener` - the listener to remove

---


### setClassifier

```
public void setClassifier(Classifier classifier)
```

:   Set the classifier to use.

    :   **Parameters:**: `classifier` - the classifier to use

---


### setDataGenerator

```
public void setDataGenerator(DataGenerator dataGenerator)
```

:   Set the data generator to use for generating new instances

    :   **Parameters:**: `dataGenerator` - the data generator to use

---


### setXAttribute

```
public void setXAttribute(int xatt)
                   throws java.lang.Exception
```

:   Set the x attribute index

    :   **Parameters:**: `xatt` - index of the attribute to use on the x axis **Throws:**: `java.lang.Exception` - if an error occurs

---


### setYAttribute

```
public void setYAttribute(int yatt)
                   throws java.lang.Exception
```

:   Set the y attribute index

    :   **Parameters:**: `yatt` - index of the attribute to use on the y axis **Throws:**: `java.lang.Exception` - if an error occurs

---


### setColors

```
public void setColors(FastVector colors)
```

:   Set a vector of Color objects for the classes

    :   **Parameters:**: `colors` - a `FastVector` value

---


### setPlotTrainingData

```
public void setPlotTrainingData(boolean pg)
```

:   Set whether to superimpose the training data
    plot

    :   **Parameters:**: `pg` - a `boolean` value

---


### getPlotTrainingData

```
public boolean getPlotTrainingData()
```

:   Returns true if training data is to be superimposed

    :   **Returns:**: a `boolean` value

---


### getColors

```
public FastVector getColors()
```

:   Get the current vector of Color objects used for the classes

    :   **Returns:**: a `FastVector` value

---


### replot

```
public void replot()
```

:   Quickly replot the display using cached probability estimates

---


### addTrainingInstanceFromMouseLocation

```
public void addTrainingInstanceFromMouseLocation(int mouseX,
                                                 int mouseY,
                                                 int classAttIndex,
                                                 double classValue)
```

:   Adds a training instance to our dataset, based on the coordinates of the mouse on the panel.
    This method sets the x and y attributes and the class (as defined by classAttIndex), and sets
    all other values as Missing.

    :   **Parameters:**: `mouseX` - the x coordinate of the mouse, in pixels.: `mouseY` - the y coordinate of the mouse, in pixels.: `classAttIndex` - the index of the attribute that is currently selected as the class attribute.: `classValue` - the value to set the class to in our new point.

---


### removeAllInstances

```
public void removeAllInstances()
```

:   Deletes all training instances from our dataset.

---


### removeTrainingInstanceFromMouseLocation

```
public void removeTrainingInstanceFromMouseLocation(int mouseX,
                                                    int mouseY)
```

:   Removes a single training instance from our dataset, if there is one that is close enough
    to the specified mouse location.

---


### startPlotThread

```
public void startPlotThread()
```

:   Starts the plotting thread. Will also create it if necessary.

---


### addMouseListener

```
public void addMouseListener(java.awt.event.MouseListener l)
```

:   Adds a mouse listener.

    :   **Overrides:**: `addMouseListener` in class `java.awt.Component`

---


### getMinXBound

```
public double getMinXBound()
```

:   Gets the minimum x-coordinate bound, in training-instance units (not mouse coordinates).

---


### getMinYBound

```
public double getMinYBound()
```

:   Gets the minimum y-coordinate bound, in training-instance units (not mouse coordinates).

---


### getMaxXBound

```
public double getMaxXBound()
```

:   Gets the maximum x-coordinate bound, in training-instance units (not mouse coordinates).

---


### getMaxYBound

```
public double getMaxYBound()
```

:   Gets the maximum x-coordinate bound, in training-instance units (not mouse coordinates).

---


### main

```
public static void main(java.lang.String[] args)
```

:   Main method for testing this class

    :   **Parameters:**: `args` - a `String[]` value


---


|  |  |  |
| --- | --- | --- |
| **Overview** \| **Package** \| **Class** \| **Tree** \| **Deprecated** \| **Index** \| **Help** \| **Weka's home** | |  |
| PREV CLASS   **NEXT CLASS** | **FRAMES**    **NO FRAMES**     **All Classes** |  |
| SUMMARY: NESTED \| FIELD \| CONSTR \| METHOD | DETAIL: FIELD \| CONSTR \| METHOD |  |


---
